# Direct and Conceptual Replications of Burgmer & Englich (2012): Power May Have Little to No Effect on Motor Performance

**DOI:** 10.1371/journal.pone.0140806

**Published:** 2015-11-04

**Authors:** Margaret Cusack, Nadya Vezenkova, Christopher Gottschalk, Robert J. Calin-Jageman

**Affiliations:** Department of Psychology, Dominican University, River Forest, Illinois, United States of America; Purdue University, UNITED STATES

## Abstract

Burgmer and Englich (2012) have reported that manipulating feelings of power can substantially improve performance on two motor tasks: golf and darts. We conducted two high-powered direct replications of the effects of power on golf, two online conceptual replications using mirror-tracing as a performance measure, and an additional conceptual replication using a cognitive performance measure (word-search). Overall, we found little to no effect of power on motor skill (*d* = 0.09, 95% CI[-0.07, 0.22], *n* = 603). We varied task difficulty, re-analyzed data without participants showing weak responses on manipulation checks, and tried adjusting performance scores for age, gender, and initial task skill. None of these secondary analyses revealed a strong effect of power on performance. A meta-analysis integrating our data with Burgmer & Englich leaves open the possibility that manipulating power could provide a modest boost in motor skill (*d* = 0.19, 95% CI [0.001, 0.38], *n* = 685). Unfortunately, the pattern of performance changes we observed was unrelated to group differences in perceived and rated power, suggesting that what motor effects do occur with this protocol may not be directly related to the construct of power. [Burgmer, P., &Englich, B. (2012). Bullseye!: How Power Improves Motor Performance. *Social Psychological and Personality Science*, 4(2), 224–232.]

## Direct and conceptual replications of Burgmer & Englich (2012): Power may have little to no effect on motor performance

“Power tends to corrupt” according to both conventional wisdom and the late moralist John Acton [1]. A recent report from Burgmer and Englich [2], however, suggests a more positive aspect of power: participants primed to feel powerful performed substantially better at both golf (Experiment 1) and darts (Experiment 2). Thus, power may put your immortal soul in danger, but it could make you great at pub games.

The work of Burgmer and Englich[2] is part of a growing body of work on the psychological effects of feeling powerful, the perception that one is at liberty to control resources as well as the behavior of others. Work in this strain has shown that priming participants to feel powerful can increase their likelihood to take action [3], reduce social perspective-taking [4], and much more. The finding that power can improve motor skills, however, stands out in two respects. First, it suggests a strong practical benefit for encouraging feelings of power (though see also [5] and others). Second, the effects reported by Burgmer and Englich are large, suggesting that control participants have a substantial reservoir of untapped motor skill which can be activated through power. Specifically, effect sizes expressed as Cohen’s *d* were 0.71 (Experiment 1, golf) and 0.73 (Experiment 2, darts).

**Fig 1:**
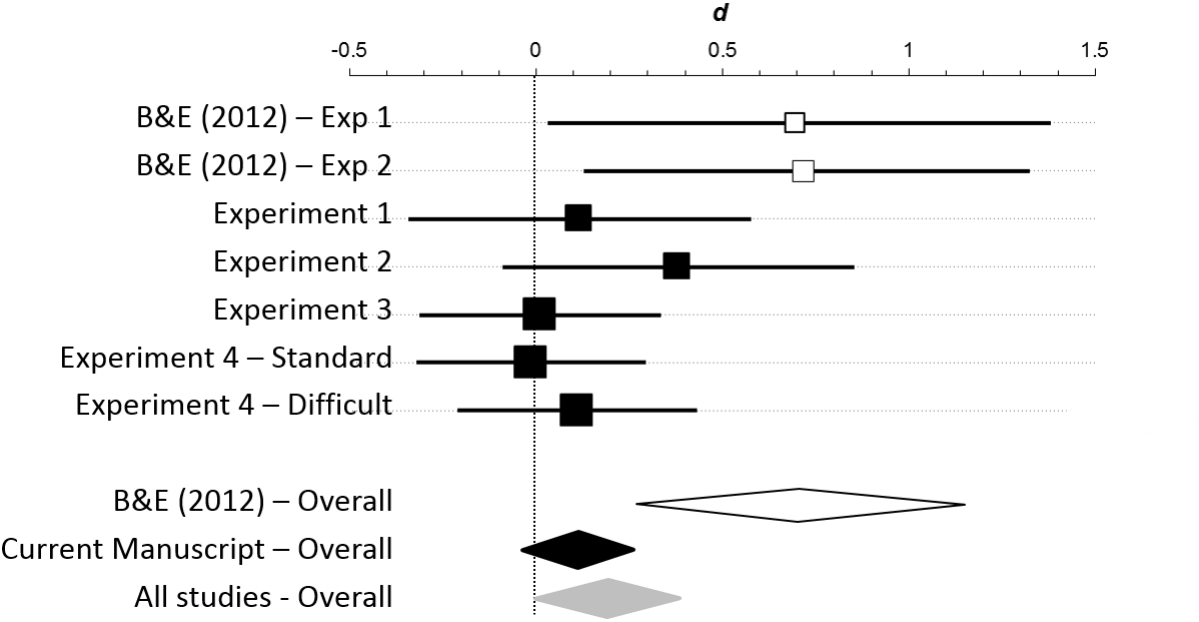
Meta-analysis of the effects of power on performance. At top are shown results the two experiments in Burgmer & Englich [2] (B&E (2012)–white squares) and from Experiments 1–4 from this paper (black squares). For each experiment, the unbiased effect size is represented by a square scaled to reflect proportionate sample size. The 95% confidence interval for the effect size is represented by a horizontal line. At bottom is shown the overall weighted effect sizes across Burgmer & Englich’s two studies (white diamond), Experiments 1–4 from this paper (black diamond), and across all studies (grey diamond). The center of the diamond represents the estimated effect size, the horizontal span of the diamond represents the 95% confidence interval for that effect. This figure was generated with the Meta-Analysis module in the Exploratory Software for Confidence Intervals package for Microsoft Excel (Cumming, 2012).

Although intriguing, the results of Burgmer & Englich[2] must be interpreted with caution due to considerable uncertainty about how the effect sizes observed might generalize to a broader population. For example, the 95% CI for the effect size from the golf study is [0.03, 1.38] (Fig 1). The data are thus consistent with the notion that power has enormous effects on performance, but are also consistent with the notion that power has vanishingly small effects on performance. The goal of this study is to provide more clarity into how strongly power affects performance. For this purpose, replication is the most suitable strategy.

To better estimate the degree to which power affects motor performance we conducted a series of direct and conceptual replications of Burgmer and Englich’s[2] golf and power experiment. Each attempt was conducted as faithfully as possible to the original protocol and with sample sizes sufficient enough to ensure strong experimental power. All data and materials are posted on the Open Science Framework: https://osf.io/d28kv/. We report how we determined our sample size, all data exclusions (if any), all manipulations, and all measures in the study [6]. Moreover, this manuscript reports all studies we have conducted on the relationship between power and performance.

## Experiment 1: Direct Replication of the Power and Golf Study

In our first replication attempt, we attempted to precisely follow the methods of Burgmer and Englich[2]. To ensure strong power for the experiment, we set a goal of recruiting at least 33 participants per group (66 total). This target is sufficient to confer power of 0.8 [7] given the effect size of 0.71 previously obtained with this experimental protocol. This study was conducted during the 2012–13 academic year and was not pre-registered

### Methods: Experiment 1

All studies reported in this manuscript were reviewed and approved by the Dominican University IRB board prior to data collection: IRB #13–02. All participants proved written informed consent, as approved in our IRB application.

#### Participants

We exceeded our sampling goal by collecting data from 73 participants from the undergraduate and post-baccalaureate populations at a private comprehensive University (55 females, 17 males, 1 not reported). This total does not include two participants who were excluded for failing to follow instructions.

Ages ranged from 18 to 27 years old (*M* = 20.4, *SD* = 2.1). Participants received research participation vouchers which they could redeem in psychology courses as part of course and extra credit assignments. In addition, all participants were given 8 pieces of candy (see below).

#### Materials

All materials were adapted as precisely as possible from Burgmer and Englich [2] except that the study was conducted in English with U.S participants rather than in German with participants in Germany. Any other deviations are noted.

In order to manipulate power, participants in the power condition were asked to recall and describe a personal memory where they felt powerful over someone else. Participants in the control condition were asked to recall and describe a situation from the previous day in which they interacted with another person. This was an open-ended question and each participant had 3 minutes to write out their memory on a piece of paper provided to them. This priming procedure was originally developed by Galinsky et al. [3].

A golf-putting task was used to measure performance. Participants were asked to perform 2 practice putts followed by 8 experimental putts from a distance of 150 centimeters. For motivation, all participants were told that they could have one piece of candy for every successful shot they make. At the end of the experiment, all participants were offered 8 pieces of candy regardless of their performance.

As a manipulation check, participants self-reported their perceived power during the recalled memory. Specifically, participants were asked “Think back to the beginning of this experiment when you were asked to recall a memory. How much did you feel in charge of the situation you described earlier?” While Burgmer & Englich[2] had participants record responses on a scale from 0 to 8, we used a scale ranging from 1 (*not at all in charge)* to *5* (*absolutely in charge)*. This deviation was not intentional, but was a mistake made by copying the scale range used for the first experimental control question (see just below).

To ensure experimental control, three questions regarding prior experience with miniature golf were used. Participants were asked to estimate how often they play miniature golf (1 = *almost never*, 5 = *more than several times per week)*, the last time they played it (1 = *about a year ago*, 4 = *more than a year ago*), and how well they thought they could play this game (1 = *not at all*, 8 = *very well*). Finally, participants were asked to self-report their age, gender, and major.

#### Procedure

Experimenters (MC and NV) memorized and rehearsed an experimental script to avoid experimenter bias and ensure equal treatment for both groups. Participants were run individually in a lab setting.

#### Ratings of Power

As an additional manipulation check we adopted the method developed by Galinsky et al. [3] for analysis of responses on the memory task. Specifically, responses were reviewed by two raters blind to both the experimental conditional and the research hypothesis. Each rater scored each essay on a scale from 1–8 for the degree of power exhibited by the narrator. Each rater was provided with a definition of power stating: “Power is defined as how capable a person believes they are in terms of controlling outcomes for oneself and others” [8].

We had not originally planned on using this additional manipulation check, but adopted it to try to understand better why the self-rating of power showed such small group differences (see below). By the time we adopted this approach, however, some of the memory response sheets had become detached from the group assignment and scoring sheets. This led to an inability to score 7 of the 73 responses (5 power and 2 control). Inter-rater reliability was strong (α = 0.94, *n* = 66); rater’s scores were averaged for each participant.

#### Data Analysis

As in the original study, independent sample *t*-tests were used to compare group means (though see [9,10]). This was supplemented with an estimation approach by calculating 95% confidence intervals for mean differences and effect sizes [11].

### Results: Experiment 1

Did priming participants to feel powerful elevate their performance on the golf-putting task? Participants in the power group performed slightly better than those in the control group (see Table 1), but this difference did not reach statistical significance (*t*(71) = .49, *p* = 0.63).

**Table 1:** Experiment 1: Summary of Group Differences.

	Control	Power	Mean Difference	Effect Size
Measure	M(SD)	M(SD)	M [95% CI]	d [95% CI]
Golf putts made	4.05 (1.67)	4.25 (1.75)	0.20 [-0.60, 0.99]	0.12 [-0.34, 0.58]
Manipulation checks:				
Perceived Power	3.27 (1.22)	3.53 (1.16)	0.26 [-0.30, 0.81]	0.22 [-0.24, 0.68]
Rated degree of power	1.25 (0.52)	5.43 (1.04)	4.18 [3.76, 4.60]	4.83 [3.86, 5.79]
Self-reported controls:				
Golf frequency	1.35 (0.48)	1.36 (0.49)	0.01 [-0.22, 0.24]	0.02 [-0.44, 0.48]
Last golfed*	3.54 (0.69)	3.44 (0.88)	-0.10 [-0.46, 0.27]	-0.13 [-0.59, 0.33]
Golf skill	2.46 (1.04)	2.61 (1.13)	0.15 [-0.36, 0.66]	0.14 [-0.32, 0.60]
Demographics				
Age	20.4 (2.2)	20.4 (2.0)		
Gender	11% males	36% males		

*Note*: Control group n = 37, power group n = 36 for all variables except rated degree of power, for which only 31 controls and 35 power could be rated.

* Note that for “last golfed” higher numbers indicate longer times since last golfing, and thus higher numbers likely indicate lower golf experience.

The small group difference in terms of motor performance was not due to a lack of experimental control. Control and power groups rated themselves similarly on frequency of playing mini golf (*t*(71) = 0.09, *p* = 0.93), last time of playing mini golf (*t*(71) = -0.5, *p* = 0.60), and mini-golf skill (*t*(71) = 0.60, *p* = 0.55). Of these control variables, only one (self-rated mini-golf skill) was a statistically significant predictor of performance (*r* = 0.47, 95% CI [0.27, 0.63], *n* = 73). Using perceived skill as a covariate, however, did not reveal a strong effect of power on performance (group means estimated with adjustment for golf skill: *M*_Power_ = 4.19, *M*_Control_ = 4.12, *F*(1,70) = 0.06, *p* = 0.81). We also found that men performed somewhat better than women (*r* = -0.33, 95% CI [-0.52, -0.11], *p* = 0.005, *n* = 72), but using gender as a covariate did not reveal a strong effect of power on performance (group means estimated with adjustment for gender: *M*_Power_ = 4.1, *M*_Control_ = 4.3, *F*(1,69) = 0.26, *p* = 0.61).

The small impact of power on motor performance was also not due to a failure to follow directions. For the 66 respondents whose memory responses could be scored, there was a large difference in rated power (*t*(64) = 20.9, *p* < 0.0009). Only 6 participants in the power group had a rating below the scale midpoint, and no participants in the control group had a rating higher than the scale midpoint. Excluding these 6 participants (plus the 7 whose responses could not be scored), actually yielded *lower* performance in the power group (*M*_Power_ = 4.00, *M*_*Control*_ = 4.10, *t*(58) = -0.22, *p* = 0.83, 95% CI for the difference [-1.00, 0.78]). Even with this restricted sample, power to detect the effect size reported in the target study (*d* = 0.71) would remain at 0.76.

Contrary to our expectations, we found that participants in the power group did not necessarily perceive their power to be stronger than those in the control group, (*t*(71) = 0.93, *p* = 0.36, all participants included). While not the effect expected, our sample size enabled us to select only for participants who responded to the self-reported manipulation check appropriately. Specifically, we selected control participants who were below the overall average for self- reported power (<3.4 on a 1–5 scale, *n* = 20) and power participants who were above the overall average for self-reported power (> 3.4 on a 1–5 scale, *n* = 20). This selection produced an enormous disparity in self-reported power (*M*_*power*_ = 4.45, *M*_*Control*_ = 2.30, *t*(38) = 11.56, *p* < 0.009). Within this selection, however, the power group performed only modestly better than controls (*M*_Power_ = 4.35, *M*_*Control*_ = 3.95, *t*(38) = .675, *p* = 0.50, 95% CI for the difference [-0.80, 1.60]). This restricted analysis has power of 0.59 given the effect size reported by Burgmer & Englich[2].

## Experiment 2: Direct Replication with Increased Task Difficulty

In analyzing the results from our first replication attempt, we noticed that our participants scored much better on the golf task (*M*_Power_ = 4.25, *M*_Control_ = 4.05) than the participants in the original study (*M*_Power_ = 3.28, *M*_Control_ = 2.28, Burgmer&Englich[2]). The higher scores could have been due to differences in golf skill between the samples or perhaps due to differences in the ‘green speeds’ used for the golf task. In either event, this disparity of performance coupled with the lack of group effects in our experiment suggests the possibility that task difficulty could serve as a moderator for the effects of power on golf skill. We thus conducted a second replication attempt with a putting distance of 300cm, twice as long as in Burgmer and Englich[2]. This distance was selected based on pilot testing to achieve control performance similar to that reported in the original study.

To ensure the previous findings were not experimenter-driven, a different researcher (CG) collected all the data for this second study. This study was also conducted during the 2012–13 academic year and was not pre-registered.

### Experiment 2: Methods

#### Participants

We again planned to collect data from at least 66 participants, and again exceeded our sampling goal by recruiting 70 participants (27 men, 43 women). Ages ranged from 17–30 (*M* = 20.1, *SD* = 1.82).

#### Materials and Procedure

All materials were the same as in Experiment 1 except the putting distance was increased to 300cm.

#### Ratings of Power

Responses to the memory prompt were again reviewed by two raters blind to both the experimental condition and the research hypothesis (scale from 1 to 8). Memory responses for 4 participants were accidentally detached from their group assignment and score sheet, so ratings were possible for only 31 control responses and 35 power responses. Inter-rater reliability was once again strong (α = 0.92, *n* = 66); rater’s scores were averaged for each participant.

### Experiment 2: Results

Our longer putting distance did succeed in reducing scores on the task (Table 2). Again, the power group performed slightly better than the control group. This difference, however, did not reach statistical significance (*t*(68) = 1.62, *p* = 0.11).

**Table 2:** Experiment 2: Summary of Group Differences.

	Control	Power	Mean Difference	Effect Size
Measure	M(SD)	M(SD)	M [95% CI]	d [95% CI]
Golf putts made	1.71 (1.64)	2.31 (1.45)	0.60 [-0.14, 1.34]	0.39 [-0.1, 0.86]
Manipulation checks:				
Perceived power	3.23 (1.00)	3.94 (0.84)	0.71[0.27, 1.16]	0.77 [0.28, 1.25]
Rated degree of power	1.71 (1.68)	5.19 (1.77)	3.47 [2.63, 4.33]	2.02 [1.42, 2.61]
Self-reported controls				
Golf frequency	1.46 (0.56)	1.20 (0.41)	-0.26 [-0.49, -0.02]	-0.53 [-1.00, -0.05]
Last golfed*	3.57 (0.65)	3.66 (0.68)	0.09 [-0.23, 0.40]	0.13 [-0.34, 0.60]
Golf skill	1.80 (0.99)	1.89 (1.02)	0.09 [-0.40, 0.57]	0.09 [-0.38, 0.56]
Demographic Variables				
Age	19.9 (1.6)	20.2 (2.0)		
Gender	31% males	46% males		

*Note*: Control group n = 35, Power group n = 35 for all variables except rated degree of power for which only 31 controls and 35 power could be rated.

* Note that for “last golfed” higher numbers indicate longer times since last golfing, and thus higher numbers likely indicate lower golf experience.

The small difference in performance was probably not due to attenuation from extraneous variables. Groups were similar in self-reported golf-skill (*t*(68) = 0.36, *p* = 0.72) and also in time since last golfing (*t*(68) = 0.54, *p* = 0.59). The control group did report a higher frequency of playing golf compared to the power group (*t*(68) = -2.20, *p* = 0.03). However, ratings on this question did not predict performance (*r* = -0.04, 95% CI [-0.27, 0.19], *p* = 0.72, *n* = 70).

As in the first study, performance was predicted by self-reported golf skill (*r* = 0.54, 95% CI[0.35, 0.69], *p* < 0.001, *n* = 70). However, using golf-skill as a co-variate did not reveal a strong effect of power on performance (group means estimated with adjustment for golf skill: *M*_Power_ = 2.28, *M*_Control_ = 1.75, *F*(1,67) = 2.8, *p* = 0.10). In addition, we tried adjusting scores for gender, as there was a non-significant trend for men to perform better than women (*r* = -0.17, 95% CI [-0.4, 0.07], *p* = 0.18, *n* = 70). Using gender as a covariate, however, did not reveal a strong effect of power on performance (*F*(1,67) = 2.01, *p* = 0.15).

The small group difference in performance was probably not due to a failure to follow instructions. Memory responses were rated much higher for the power group compared to the control group (*t*(64) = 8.16, *p* < 0.0009). Moreover, the power group reported higher perceived power compared to the control group (*t*(64) = 3.23, *p* = 0.002). While this indicates successful manipulation of power, there were 12 participants who had memory responses that were rated contrary to group assignment (power group: 9 participants with an average rating below the scale midpoint; control group: 3 participants above the scale midpoint). Screening out these participants (as well as 4 participants whose responses which could not be rated) did not reveal an effect of power on performance; in fact this brought the group means *closer* together (*M*_Power_ = 2.01, *M*_*Control*_ = 1.82, *t*(52) = 0.59, *p* = 0.55, 95% CI for the difference [-0.61, 1.12]). Given the effect size reported in Burgmer & Englich[2], power for this restricted analysis would be 0.73.

## Experiment 3: Online Replication Using Mirror-Tracing Task

One drawback of the golf task is that it involves substantial and sustained interaction between the experimenter and the participant over the course of the task. It is possible, then, that an experimenter could subconsciously influence participant performance to bias performance in the direction of expected effects (e.g. [12]). Although we had used an experimental script to minimize such possibilities, it seemed wise to attempt a conceptual replication involving a minimum of experimenter interaction.

To this end, we developed a conceptual replication using a measure of motor performance that could be administered in an online context: mirror tracing. We selected this task because of its ease of online administration, long-standing use in the motor-skill literature, known inverse correlation with age, and possibility for examining learning as well as initial skill level (see below). Mirror tracing is quite distinct from golf, but Burmer & Englich[2] concluded from their studies that power improves motor performance without any qualification on the type of motor task.

This study was conducted over the course of 2 days in September, 2014. Prior to data collection, we pre-registered the materials, sampling strategy, and analysis plan.

### Experiment 3: Methods

#### Participants

Participants were recruited for an online study using Mechanical Turk and paid $0.50 USD for completing the study. We restricted recruitment to U.S.-based participants who had at least 80% payment rates and who had not participated in a pilot study of the mirror-tracing task (see below).

We set a goal of at least 53 participants per group, as this would provide power of at 0.95 for the effect size of 0.71 found by Burgmer & Englich[2]. As we were unsure what attrition rate to expect with the quality controls we adopted (see below), we set a generous goal of at least 200 responses. In total, 435 participants accessed the pre-screening survey (see below), 306 of these continued on to the main study, and 210 completed the study.

#### Materials and Procedure

**Fig 2:**
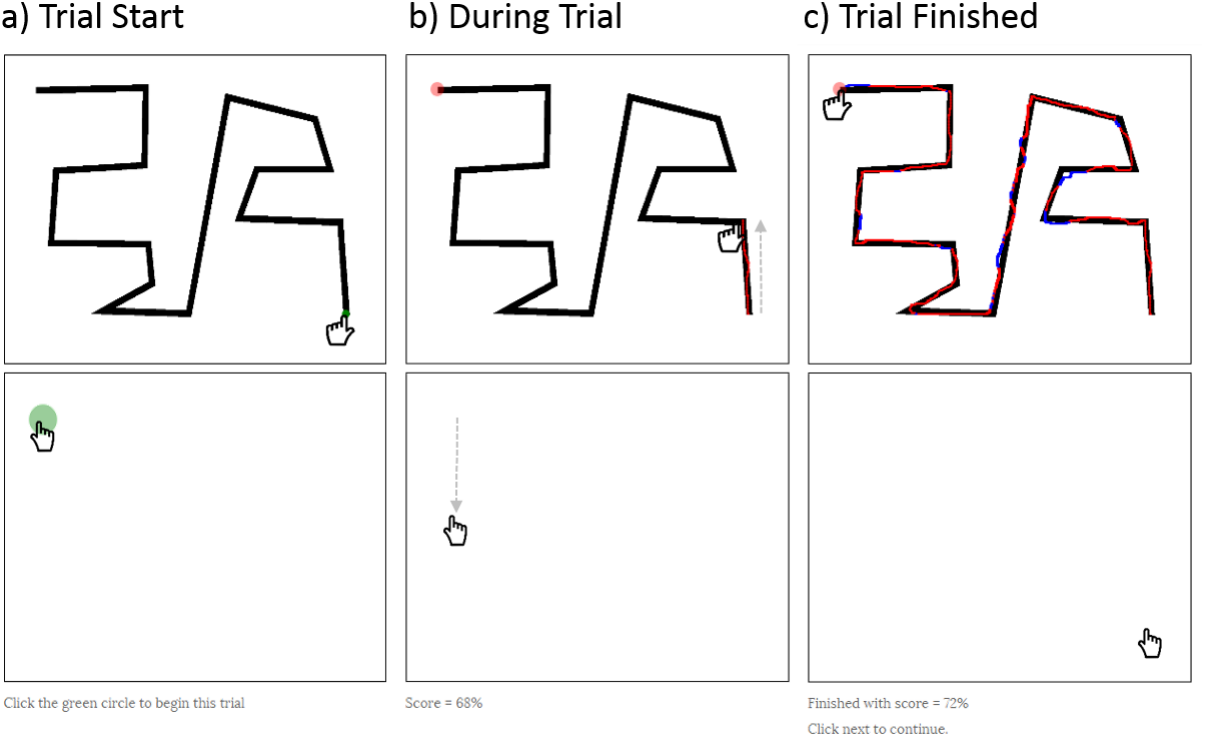
Online Mirror Tracing Task. The task displayed two rectangular canvases: the bottom canvas was the “drawing pad” where the participant actually moved the cursor; the top canvas was the “mirror” which showed an image of the figure to trace and which produced a mirrored-trail of the path of mouse movement on the drawing canvas. A) To start the task, participants moved the cursor into a green start box on the drawing pad and click the mouse. This caused the mirrored cursor to come into register with the beginning of the figure. B) Once the trial was initiated, all mouse movements within the drawing pad produced a colored, mirrored trail in the mirror. To illustrate, the dotted line shows the path of the cursor straight down in the drawing pad, the corresponding mirrored trail straight up in the mirror. The trail left is colored red when within the figure to trace, blue when within. The current score is shown just below the drawing pad. C) The trial finished when the red target is reached. In this case, the score achieved is 72%. Participants could move directly to the target, but this would yield a very low score. A demonstration of this task is posted online at: http://tinyurl.com/mirror-task; source code is posted to the Open Science Framework.

Mirror Tracing Task: As a dependent measure we developed an online mirror-tracing task using JavaScript and HTML5 (Fig 2). The display had two rectangular canvases—the top canvas was the ‘mirror’ and the bottom canvas was the ‘drawing pad’. When the participant moved the cursor within the drawing pad, it produced an inverted trail on the mirror but left no marks on the canvas. In each trial, the mirror presented a different complex line drawing, and the participant’s goal was to trace the drawing as precisely as possible within a 2-minute/trial time limit. The trial and timer would begin with the participant moving the cursor into a start-box on the drawing pad, and would conclude upon reaching an end box marked on the screen. If the end box had not been reached within 2 minutes, the trial was terminated and the current score recorded as final. In each trial, the line drawing to be traced varied, but always had the same line thickness, same number of edges, and the same total drawing distance. A demonstration of this task is posted online at: http://tinyurl.com/mirror-task; source code is posted to the Open Science Framework.

The score for each trial was calculated as the percentage of each trail within the target outline. Feedback was given by showing the current score for each trial below the drawing pad and by color-coding the trail red when within the outline and blue when outside the outline.

To validate our measure, we asked 51 Mechanical Turk participants to complete a series of 6 consecutive mirror-tracing tasks. We found that scores averaged 44% across the trials. No participant scored higher than 80%, indicating no risk of ceiling effects. Furthermore, reliability was high across trials (α = 0.94). There was evidence of improvement in scores from the first trial to the second (*t*(50) = 4.39, *p* < 0.001), after which scores were stable. As expected from prior studies with mirror-tracing tasks [13], there was a negative correlation between age and mirror-tracing performance (*r* = -0.30, 95% CI [-0.53, -0.03], *n* = 50). We found similar performance (*M* = 43% over all trials and groups) and reliability (α = 0.920) in the main experiment.

Pre-Screening Survey: We used Qualtrics to administer the study (http://qualtrics.com). Those electing to participate via Mechanical Turk were first directed to a pre-screening survey. This gave a single non-mirrored tracing trial with a 1 minute time limit. Those who achieved either no score or a score of 0% were then excluded from signing up for the study. This was done both as a warm-up and to ensure that only participants who had a compatible browser and interface device could sign up.

The pre-screening survey also contained an instructional manipulation check [14]. Specifically, participants were given a multiple choice question in which the responses were the names of different browsers (Firefox, Chrome, Internet Explorer, Safari, or Other). The actual text of the question, however, asked the participants to choose “Other” and to enter their Mechanical Turk ID. Those who did not follow the directions were returned to the same question with the prompt to read all questions carefully. The number of failures was recorded and used as a quality-control screen (see below).

Primary Measures: The main survey attempted to precisely adapt the procedure of Burgmer & Englich[2] to an online context. We used the same memory prompt (neutral or control) to manipulate power, followed by the 3-trial mirror-tracing task, and then by the same manipulation check (perceived power are rated degree (1–5) of feeling ‘in charge’ during the memory recalled).

Positive Control: As a positive control, we included a modified retrospective gambler’s task [15] after the manipulation check. In this task, participants were asked to imagine entering a casino where they observe a gambler role 3 dice and obtain either a) 3 sixes (the all-sixes scenario) or b) 2 sixes and a three (the some-sixes scenario). We obtained the materials for this task from the Many Labs project [16], and as in this study we dropped a third condition, in which the gambler rolls 2 dice and gets 2 sixes. After imagining the scenario, participants were asked to estimate how long the gambler had been rolling dice. The classically observed effect is for those who read the 3-sixes scenario to estimate more prior rolls than those who read the 2-sixes scenario.

Group assignment was made randomly and independent of memory condition. This positive control was selected because a) the Many Labs project has recently shown that this effect is highly robust [16], b) the expected effect size (*d* = 0.61) is similar to that observed for power and performance by Burgmer & Englich[2], and c) the effect depends critically on participant’s reading carefully enough to respond differently to a quite subtle difference between the two scenarios. We used this positive control both to help screen participants not engaged seriously in the study (those giving highly outlandish estimates of previous rolls, see below) and as evidence of the quality of the overall sample and procedure. We followed the Many Labs project in applying a square root-transformation to estimated rolls, but report raw scores for ease of interpretation.

Additional Measures: After the positive control, participants completed a section with basic demographics (gender, age, and ethnicity). They then entered a final section in which they were asked a) how familiar they were with the memory task (scale of 1–4), b) if they were native-English speakers, c) if they were currently living in the U.S.A., and d) what they thought the hypothesis of the study was. For this last section, participants were assured that their payment for participation was guaranteed, and they were encouraged to be completely honest in their responses.

#### Quality Controls

Although Mechanical Turk samples can provide results that are highly consistent with traditional participant pools [17], there are legitimate concerns about such samples. This includes the possibility of previous exposure to study materials [18] and low engagement [19]. To help avoid these problems, we pre-established a number of quality control filters and registered these on the Open Science Framework prior to data collection. Each filter is explained in more detail in our pre-registration plan, but the overall goal was to ensure that the participants selected would be a) fluent in English, b) engaged in the task, and c) unfamiliar with the study materials.

From the 210 complete responses collected (104 power, 106 control), we applied the following quality controls sequentially, excluding

- 8 participants (5 power and 3 controls) for using an IP address that resolved to an address outside the U.S.A,
- an additional 2 participants (1 power, 1 control) for failing the instructional manipulation check more than 2 times,
- an additional 7 participants (4 power, 3 controls) for classifying themselves as non-native speakers of English,
- an additional 29 participants (16 power, 13 controls) for self-reporting that they were already familiar with the memory prompt (>2 on rating of familiarity on scale from 1–4),
- an additional 3 participants (2 power, 1 control) for taking an unusually short or long amount of time to complete the study (outside of 2.5x the median time for completion),
- an additional 1 participant (1 controls) for giving an outlier response on the retrospective gambler’s task (|z| > 3),
- an additional 11 participants (10 power, 1 control) for guessing the hypothesis of the study (any response that mentioned anything to do with power or control in relation to performance was excluded).

In total, these filters excluded 29% of completed responses, leaving 66 participants in the power group and 83 in the control group. These exclusions did not substantively impact the group means for the mirror-tracing task. Moreover, even with all of these quality controls applied, power was strong (0.99) for detecting the effect size of 0.71 reported in Burgmer & Englich[2].

#### Ratings of Power

Responses to the memory prompt were again reviewed by two raters blind to both the experimental conditional and the research hypothesis (scale from 1 to 8). To save time, only those responses which had passed all quality controls were reviewed. Inter-rater reliability was again strong (α = 0.96). Rater’s scores were averaged for each participant.

### Experiment 3: Results

Power did not have a substantial impact on overall motor performance as assessed by the average of all 3 mirror-tracing trials (*t*(147) = 0.07, *p* = 0.95). Those in the power group stayed within the line just 0.6% more than those in the control group (Table 3). Similar results were obtained when examining trial-by-trial averages. Moreover, we assessed if power might alter learning by analyzing group differences on the third trial adjusted for first-trial scores. Contrary to expectation, there was a non-significant trend for those in the control group to improve more than those in the power group (trial 3 group means estimated with adjustment for trial 1: M_Power_ = 42%, M_Control_ = 46%, F(1,146) = 3.11, p = 0.08).

**Table 3:** Experiment 3: Summary of Group Differences.

	Control	Power	Mean Difference	Effect Size
Measure	M(SD)	M(SD)	M [95% CI]	d [95% CI]
Mirror Tracing Score	44% (23%)	44% (22%)	0.3% [-7%, 8%]	0.01 [-0.31, 0.34]
Manipulation checks:				
Perceived power	3.01(1.15)	3.68(1.23)	0.67[0.28, 1.05]	0.56 [0.23, 0.89]
Rated degree of power	1.12 (0.46)	5.95(1.49)	4.82 [4.44, 5.21]	4.36 [3.77, 4.96]
Demographic Variables:				
Age	36.5 (12.7)	33.2 (10.77)		
Gender	52% males	39% males		

*Note*: Control group n = 83, Power group n = 66.

The failure to observe a strong effect of power on motor performance was not due to insufficient manipulation, as there was a clear impact of condition on perceived power (*t*(145) = 3.38, *p* = 0.001). Moreover, independent ratings of power expressed in the memory response showed a clear group difference (t(73.8) = 25.13, *p* < 0.001). Despite this, there were 8 participants who gave memory responses that were rated contrary to group assignment (7 in the power group with responses rated below the scale midpoint; 1 in the control group with a response rated above the scale midpoint). Removing these participants, however, did not reveal a strong impact of power on performance (*M*_Power_ = 44%, *M*_*Control*_ = 43%, *t*(139) = 0.07, *p* = 0.95). Given the effect size observed in the target study, power was 0.98 in this restricted analysis.

**Fig 3:**
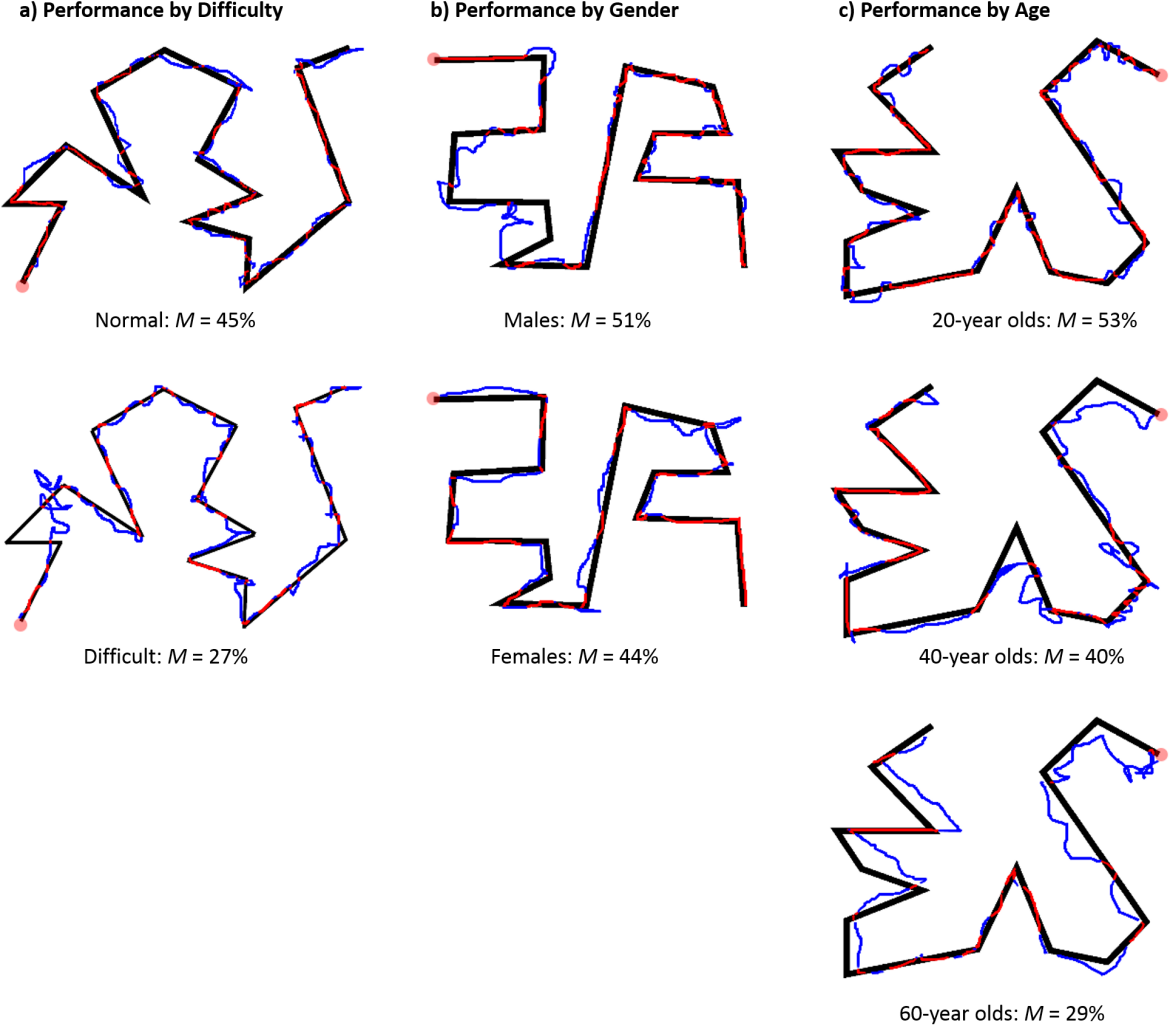
Examples of mirror tracing performance for different difficulties, genders, and ages. Each tracing is from an actual participant in Experiment 4. Tracings were selected to have a score at the mean for the category. A) Task difficulty: reducing the line thickness of the tracing form substantially increased difficulty and reduced scores. B) Gender: Men tended to perform slightly better than women on this task. C) Age: Younger participants were somewhat better at this task than older participants.

Consistent with prior literature on motor skill (reviewed in [13]), we found that older participants performed worse on mirror-tracing than younger participants (*r* = -0.34, 95% CI [-0.48, -0.19], *p* < 0.001, *n* = 149, Fig 3C). In addition, we found a near-significant advantage for men relative to women (*r* = -0.16, 95% CI [-0.31, 0.01], *p* = 0.06, *n* = 149, Fig 3B). Using age and gender as covariates, however, did not reveal a benefit of power on motor performance (group means estimated with adjustment for age and gender: M_Power_ = 42%, M_Control_ = 45%, F(1,145) = 0.59, p = 0.45).

For the positive control, results were in the expected direction. Specifically, those asked the “all-six” question on the retrospective Gambler’s fallacy reported higher estimates than those asked the “two-sixes” question (*t*(147) = 1.99, *p* = 0.049). This effect, though, was weaker than expected, indicating that there may have been some problems with task engagement.

## Experiment 4: Online Replication with Alternative Manipulation

One problematic finding in our online replication is the high degree of familiarity Mechanical Turk participants have with the autobiographical memory task as a manipulation of power. Specifically, 14% of all respondents reported that they were either familiar or very familiar with this task. Although we excluded these participants from our analysis, it is possible that additional participants had experienced the task but did not elect to report it. Prior familiarity with a manipulation or measurement is problematic, as it can greatly attenuate effects observed in naïve participants [20]. Therefore, we sought to conduct an additional conceptual replication of Burgmer & Englich[2] using a manipulation that would be less familiar to Mechanical Turk participants. To this end, we developed an online version of the manipulation in Experiment 2 of Burgmer & Englich[2], the power-priming word-search task created by Chen, Lee-Chai and Bargh [21]. We used Google scholar to search papers citing Chen et al. [21]). Although this approach has proven very popular for manipulating power, we did not find any prior *published* work using the word-search task to manipulate power with a Mechanical Turk sample. We thus hypothesized that this task would be fairly unfamiliar to Mechanical Turk participants.

In this study we also explored the effect of task difficulty. Half the participants completed the same 3 mirror-tracing figures used in Experiment 4. The other half, however, completed the same shapes with line-thickness reduced by ~50%. Reduced line thickness provides a smaller target for tracing, and was expected to make the task more challenging (Fig 3A).

This study was conducted over the course of 7 days in Feburary, 2015. Prior to data collection, we pre-registered the materials, sampling strategy, and analysis plan.

### Methods: Experiment 4

#### Participants

Participants were again collected via Amazon’s Mechanical Turk and paid $0.50 USD for completing the study. We posted requests for 450 completions. In total, 968 workers accessed the pre-survey screen, 474 of these continued on to the main study, and 442 provided complete responses. From these, we implemented a pre-registered set of quality controls similar to those described in Experiment 3, excluding:

- 21 participants (12 power and 9 control) for using an IP address that resolved to an address outside the U.S.A,
- an additional 10 participants (6 power, 4 control) for failing the instructional manipulation check more than 2 times,
- an additional 15 participants (6 power, 9 control) for classifying themselves as non-native speakers of English,
- an additional 62 participants (36 power, 26 control) for self-reporting that they were already familiar with the word-search task (>2 on rating of familiarity on scale from 1–4, where 1 = *Not familiar*, *I’ve never completed an online word search before*, *2 = Somewhat familiar*, *I’ve completed word searches like this online before*, *but not with that word list*, *3 = Familiar*, *I’ve completed word searches like this online before*, *and some of the words were probably the same*, *4 = Very familiar*, *I’ve completed an online word search before using this exact same word list*)
- an additional 3 participants (3 power) for taking an unusually short or long amount of time to complete the study (outside of 2.5x the median time for completion),
- an additional 1 participant (1 control) for reporting a technical problem with the mirror-tracing task
- an additional 4 participants (3 power, 1 control) for reporting a valid reason to not use their data (e.g. “distracted” or problems viewing the word search)
- an additional 9 participants (4 power, 5 control) for solving less than 7 of the 10 words on the word search
- an additional 5 participants (1 power, 4 control) for doing very poorly on the mirror-tracing task (<5% averaged over all 3 trials)
- no additional participants for guessing the hypothesis of the study (any response that mentioned anything to do with power or control in relation to performance was excluded)

In total, these filters excluded 29% of completed responses, leaving 164 controls (81 standard task, 83 difficult) and 148 power (80 standard task, 68 difficult). Contrary to our expectations from the currently published literature; familiarity with the word-search task was self-reported to be as familiar on Mechanical Turk as the autobiographical memory task (14% of all completed responses rated the word-search task as familiar).

#### Materials and Procedure

The materials were the same as in Experiment 3 except that the manipulation was changed to a word-search task (see below) and the mirror-tracing task was presented with 3 normal-difficulty figures (same as in Experiment 3) or 3 difficult figures (same shapes as in Experiment 3 but with line thickness reduced by ~50%). The positive control was presented as before, but due a glitch all participants were assigned the all-sixes scenario. Thus, we were unable to analyze data from this task.

**Fig 4:**
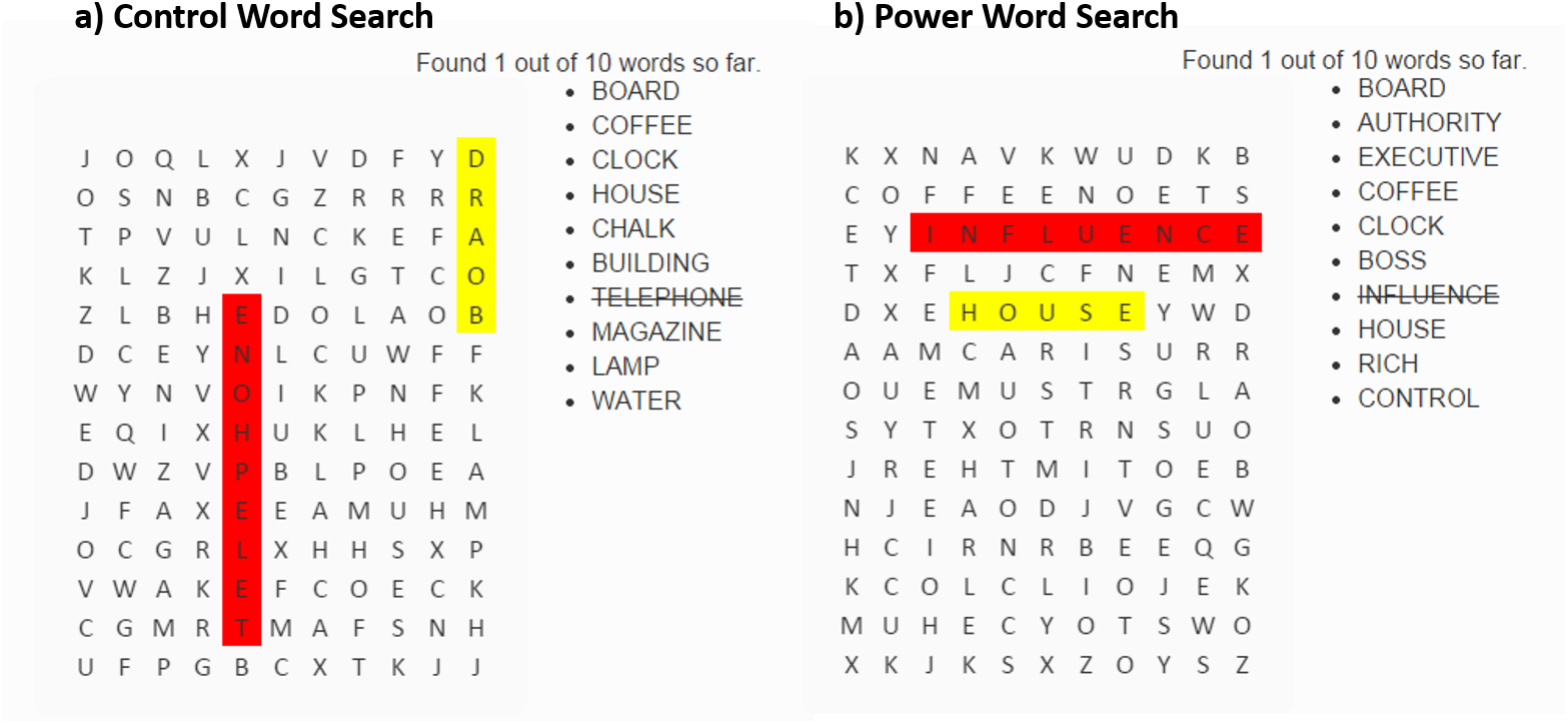
Word Search Manipulation of Power. A) Grid and word list used for the control condition; b) Grid and word list used for the power condition. These are the same grids and lists as used in Gravelin [22]; the task was first introduced by Chen et al. [21]. A demonstration of this task is posted online at: http://tinyurl.com/word-search-task; source code is posted to the Open Science Framework.

Word Search Task: To manipulate power, we adapted the word-search priming task developed by Chen et al. [21] for online presentation using JavaScript and HTML5 (Fig 4). A demonstration is posted to http://tinyurl.com/word-search-task; source code is available on the Open Science Framework.

Participants were presented with a 14x11 grid of letters and a word-bank of 10 words to find. For half the participants, the word bank had 6 words related to power (*authority*, *executive*, *boss*, *influence*, *rich*, *control*) and 4 neutral words (*board*, *coffee*, *clock*, *house)*. For the other half of the participants, the word bank had 10 neutral words (*board*, *coffee*, *clock*, *house*, *chalk*, *building*, *telephone*, *magazine*, *lamp*, *water*). The exact word list and grid were taken from the appendix of Gravelin [22].

Participants highlighted words by clicking a letter and dragging over the word. When a highlighted selection matched an item in the word bank, that part of the grid was highlighted in red and the corresponding item in the word-bank was marked complete by presenting it in strike-through font. During the word search, the “Next” button was de-activated, forcing participants not to skip the task. The survey continued only when a) all 10 words had been found or b) 5 minutes had elapsed. In either case, the survey would then proceed automatically to the next task.

### Experiment 4: Results

As expected, reduced line thickness made the mirror-tracing task more difficult, producing a large reduction in scores (*F*(1,308) = 78.2, *p* < 0.0009). We did not observe, however, a strong effect of power on performance (*F*(1,308) = 0.1, *p* = 0.75) nor an interaction between difficulty and power on performance (*F*(1,308) = 0.21, *p* = 0.65). Results are summarized in Table 4. In the standard difficulty condition, scores in the power condition were very slightly lower than in the control condition (*t*(159) = -0.08, *p* = 0.93). In the enhanced difficulty condition, the power group stayed within the line 1.6% more often than in the control group (*t*(149) = 0.67, *p* = 0.50). Although scores improved after the first trial, power did not strongly relate to changes in motor skill across the experiment (*F*(1,307) = 0.10, *p* = 0.76 for main effect of power; *F*(1,307) = 0.01, *p* = 0.92 for interaction of power and difficulty on third-trial scores adjusted for first trial scores).

**Table 4:** Experiment 4: Summary of Group Differences.

	Control	Power	Mean Difference	Effect Size
Measure	M(SD)	M(SD)	M [95% CI]	d [95% CI]
Mirror Tracing Score–Standard	50% (21%)	50% (22%)	0.3% [-6%, 7%]	-0.01 [-0.32, 0.30]
Mirror Tracing Score–Difficult	31% (15%)	32% (15%)	1.6% [-3%, 6%]	0.11 [-0.21, 0.43]
Demographic Variables:				
Age	32.8 (12.1)	33.2 (10.8)		
Gender	42% males	55% males		

*Note*: For standard mirror-tracing task: Control group *n* = 81, Power group *n* = 80. For difficult mirror tracing task: Control group *n* = 83, Power group *n* = 68.

As expected, mirror-tracing skill was negatively correlated with age (*r* = -0.29 95% CI[-0.39, -0.19], *n* = 311) and men performed better than women (*r* = -0.22 95% CI[-0.32, -0.11], *n* = 312). Using age and gender as covariates, however, did not reveal a strong impact of power on performance (*F*(1,305) = 0.02, *p* = 0.88 for main effect of power; *F*(1,305) = 0.09, *p* = 0.76 for interaction of power and difficulty). Applying an even stricter criterion for familiarity (only those who rated familiarity as a 1 on a 4-point scale) reduced sample size to 176 participants but did not alter the pattern of results in any substantive way.

## Experiment 5: Effect of Power on a Cognitive Task

Burgmer & Englich [2] proposed that power enhances motor performance by increasing goal pursuit. This prediction drew, however, from extensive prior work on the cognitive effects of power, which suggests that the enhanced goal pursuit produced by feelings of power can accelerate task selection and increase attentional focus on task-relevant goals [23,24]. Thus, we also examined if priming for power could influence performance on a cognitive task. As a dependent measure, we selected a word-scramble in which participants make as many words as possible from a pool of letters within a set period of time. We selected this task because it seemed reasonable to expect that stronger goal pursuit, faster goal selection, and better attentional skills could aid in this type of skill. Furthermore, this task had previously been reported to exhibit large group effects with a subtle manipulation [25], though this effect has since been shown to not be easily replicable [26].

### Methods: Experiment 5

#### Participants

This was an in-person study conducted with university students drawn from our introduction to psychology participant pool. We again exceed the sample size estimated to provide strong power (33/group for power of 0.80) by collecting data from 90 participants (70 females, 20 males). This total does not include two participants excluded for not following instructions. Ages ranged from 17–28 (*M* = 20, *SD* = 1.78).

#### Materials

We manipulated power with the same autobiographical memory task used in Experiment 1 of Burgmer & Englich [2].

As a dependent measure, we used a word-scramble task adapted from Damisch et al. [25]. Specifically, participants were given a set of scrambled letters to form words from (RSTLNEAP). The time allotted to complete the task was 3 minutes and participants were only allowed to use the letters in the set, with each letter being used only once. The total number of rule-abiding words generated was used as an indicator of performance. Two experimenters (MC and NV) scored all responses.

#### Procedure

For this experiment, participants were run in groups, usually in a classroom setting. Group assignment was randomized by handing out packets with different instructions (power or control) for the memory task. After completing the power manipulation, participants turned the page of their packet to the word scramble task. Participants thus had minimal interactions with the experimenter, and both conditions were run simultaneously ensuring strong experimental control.

#### Ratings of Power

The same two raters scored all memory responses, remaining blind to experimental condition and the research hypothesis. Inter-rater reliability was strong (α = 0.92, *n* = 90).

### Experiment 5: Results

The power group performed better than the control group (Table 5). This difference, however, did not reach statistical significance (*t*(70.33) = 1.37, *p* = .18). The power group had substantially higher variance, due primarily to the presence of an outlier who generated 76 words (z = 4.5). Neither gender nor age were significant predictors of performance on this task.

**Table 5:** Experiment 5: Summary of Group Differences.

	Control	Power	Mean Difference	Effect Size
Measure	*M(SD)*	*M(SD)*	*M [95% CI]*	*d* [95% CI]
Words Created	21.61 (7.72)	24.90 (14.13)	3.26 [-1.55, 8.06]	0.29 [-0.21, 0.43]
Manipulation checks:				
Perceived power	3.57 (0.73)	3.59 (1.05)	0.02 [-0.36, -0.40]	0.02 [-0.39, 0.43]
Rated degree of power	1.25 (0.52)	5.35 (1.50)	4.10 [3.62, 4.57]	3.65 [2.96, 4.32]
Demographic Variables:				
Age	20.3 (1.9)	20.2 (1.7)		
Gender	25% males	20% males		

*Note*: Control group n = 44, Power group n = 46.

Ratings of power responses revealed that participants had, indeed, written about divergent experiences (*t*(88) = 17.14, *p* < 0.009). Nevertheless, 12 participants in the power group gave responses rated contrary to condition (<5 on a scale from 1–8). Removing these participants, however, did not reveal a strong effect of power on performance but instead brought the group means closer together (*M*_Power_ = 24.4, *M*_*Control*_ = 21.6, *t*(50.0) = 1.10, *p* = 0.28, 95% CI for the difference [-2.3, 7.9]).

Despite the difference in rated power, we observed a surprising lack of difference in perceived power (*t*(88) = 0.98, *p* = .92). We thus screened specifically for participants who exhibited group-appropriate responses to this question. Control participants were selected who were below the overall average for self- reported power (<3.5 on a 1–5 scale, n = 21), and power participants were selected who were above the overall average for self-reported power (>3.5 on a 1–5 scale, *n* = 31). This selection produced a distinct difference in feeling ‘in charge’ (*M*_*power*_ = 4.2, *M*_*control*_ = 2.9, *t*(50) = 12.29, *p* < 0.009). However, within this selection, primed participants performed only modestly better than controls (*M*_power_ = 25.5, *M*_control_ = 21.2, *t*(46.2) = 1.13, *p* = 0.27, 95% CI for the difference [-3.34, 11.90]). This restricted analysis has power of 0.69 given the effect size reported by Burgmer & Englich [2].

## Relationships

We explored the relationships between performance, perceived power, and rated power across experiments 1, 2, 3, and 5. Experiment 4 was not included in this analysis because it used a word-search prime that was not amenable to conducting manipulation checks. To enable comparisons across studies, performance was expressed in standardized values relative to each experiment.

Across all studies and both conditions, perceived power was weakly related to performance (*r* = 0.13, 95% CI[0.03, 0.23], *n* = 368). Note, however, that perceived power was measured *after* performance. Rated degree of power was not related to performance (*r* = 0.03, 95% CI[-0.07, 0.13], *n* = 370). Oddly, rated and perceived power were only moderately correlated (*r* = 0.22, 95% CI[0.12, 0.31], *n* = 368).

## Meta-analysis

We conducted a meta-analysis on the effect of power on motor performance using the ESCI software package for Microsoft Excel [11]. All results are reported using a random effects model.

For the original results (golf and darts) of Burgmer & Englich [2] we found an overall unbiased estimate of effect size (Cohen’s *d*) of 0.71, 95% CI [0.27, 1.15], equivalent to an increase from the 50^th^ to 76^th^ percentile. This is shown in Fig 1 as a white diamond.

From our four studies on motor performance (experiments 1–4), we achieve a considerably smaller estimate: *d* = 0.09, 95% CI[-0.07, 0.22], equivalent to an increase from the 50^th^ to the 54^th^ percentile and consistent with the possibility of no effect. This is shown in Fig 1 as a black diamond. Note that our estimate has no overlap with what was obtained in the original study.

Integrating across both manuscripts provides an intermediate estimate of 0.19, 95% CI [0.001, 0.38], equivalent to an increase from the 50^th^ to the 57^th^ percentile. This is shown in Fig 1 as a grey diamond. Note, that this integrated estimate does *not* show significant heterogeneity of effect size (*Q*(6) = 9.0, *p* = 0.17). This occurred because of the trend in our data towards an effect and also because the data from Burgmer and Englich [2] are compatible with a very wide range of effect sizes. The integrated effect size suggests the potential for a small effect of power on performance, but the confidence interval is consistent with an effect that is moderate down to vanishingly small. In addition, even this integrated effect size estimate is not statistically consistent with what was obtained in the original study.

We did not include our Experiment 5 in the meta-analysis because it the dependent variable is so different. Including this study as well, however, would not substantially revise the overall effects size shown in Fig 1 (*d* = 0.20, 95% CI[0.03, 0.36]).

**Fig 5:**
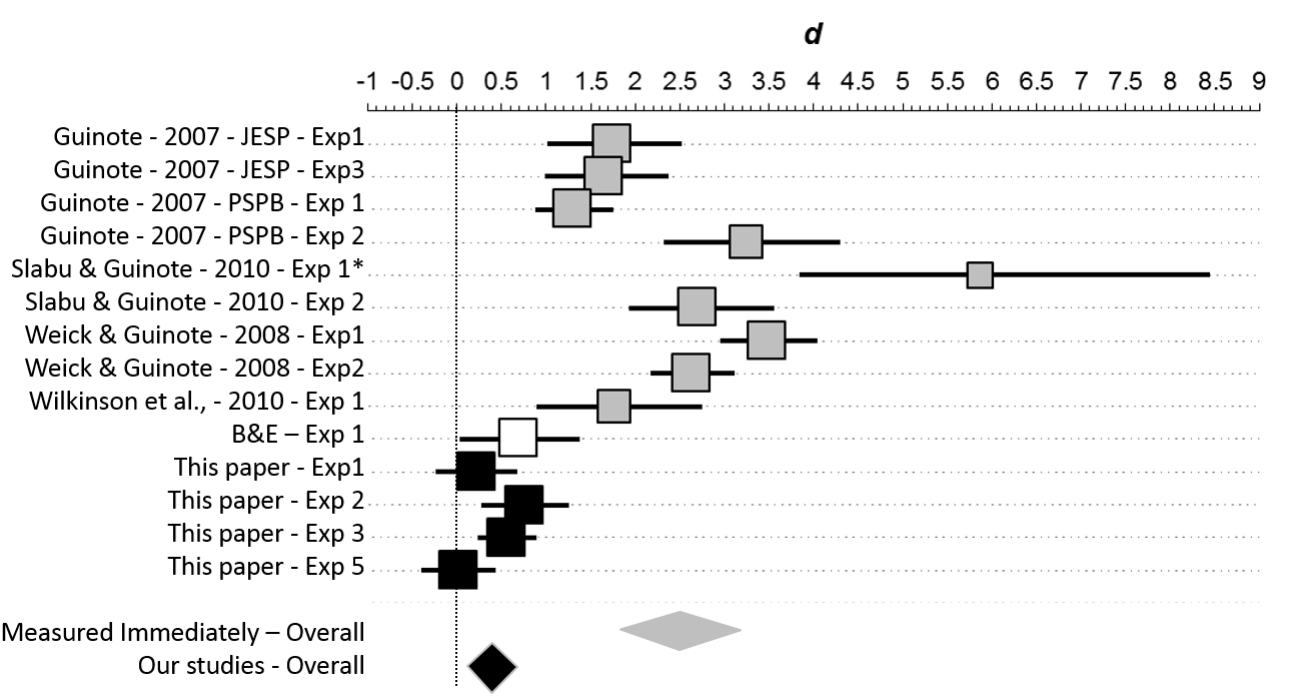
Meta-analysis of the effects of the autobiographical memory task on perceived power. We included our study, Experiment 1 from Burgmer & Englich (2012) (experiment 2 did not contain a perceived-power manipulation check) and all studies we could easily locate which used a perceived power manipulation check after using the autobiographical memory task to manipulate power. For each experiment, the unbiased effect size is represented by a square scaled to reflect proportionate sample size. The 95% confidence interval for the effect size is represented by a horizontal line. Studies colored grey measured perceived power immediately after the manipulation, and their overall effect size is represented by the grey diamond (*d* = 2.5, 95% CI [1.9, 3.2]). Burgmer & Englich (Experiment 1, 2012) moved this manipulation check to the end of the study, after the performance measure, and obtained a much smaller effect on perceived power (white square: *d* = 0.7, 95% CI [0.03, 1.4]). Our studies (shown in black) followed the sequence from Burgmer & Englich (2012). Only our Experiment 5 has an effect size (barely) outside the CI from Burgmer & Englich (2012) and our overall effect size (black diamond) is not radically dissimilar: *d* = 0.4, 95% CI [0.07, 0.7]. Significant heterogeneity of effect size is evident, even within the restricted set of studies that conducted the manipulation check immediately after the manipulation (*Q*(8) = 63.5, *p* < 0.009). All effect sizes from prior literature were calculated based on reported *t* and *df* values. This figure was generated with the Meta-Analysis module in the Exploratory Software for Confidence Intervals package for Microsoft Excel (Cumming, 2012). * In Slabu & Guinote (2010) Experiment 1 reports “t(17) = 127.41”, which yields *d* = 61.8. The summary data reported, however, are consistent with an effect size of about 6.1. We suspect, then, that the t value provided had a decimal place transposed, and used *t*(17) = 12.74, yielding *d* = 6.18. We used this more plausible value in this meta-analysis. Note, though, that even this value stands out as an exceptionally strong effect relative to other studies reported in the literature.

We also used ESCI to conduct a provisional meta-analysis of the effect of the autobiographical memory task on perceived power (Fig 5). For perceived power, previous reports [23,24,27–29] administered the manipulation check immediately after the autobiographical memory test and obtained very strong group differences (*d* = 2.5, 95% CI [1.9, 3.2]). Burgmer & Englich [2] administered the perceived-power manipulation check after the performance measure, and obtained a substantially smaller effect (Experiment 1: *d* = 0.7, 95% CI [0.03, 1.4]). Our studies used the same sequence and found an effect even smaller (*d* = 0.4, 95% CI [0.07, 0.7]) but not dramatically different from Burgmer & Englich [2].

**Fig 6:**
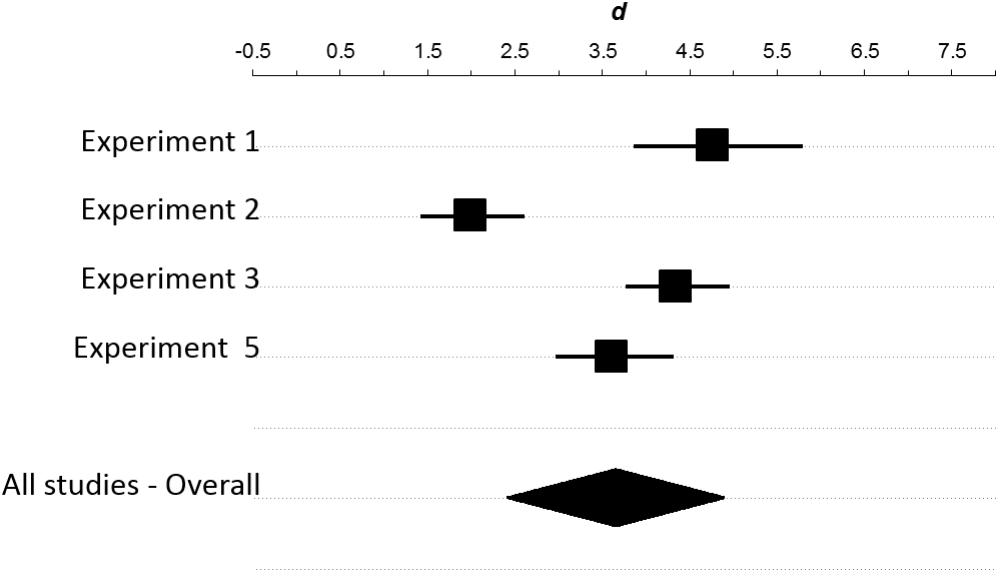
Integration of rated-power manipulation check results across the studies reported in this manuscript. This is not a comprehensive meta-analysis, but simply an integration of results to evaluate consistency and overall effect size. For each experiment, the unbiased effect size is represented by a square scaled to reflect proportionate sample size. The 95% confidence interval for the effect size is represented by a horizontal line. At bottom is shown the overall weighted effect sizes across all studies (black diamond). The center of the diamond represents the estimated effect size, the horizontal span of the diamond represents the 95% confidence interval for that effect. For this analysis, the overall estimate is: d = 3.7, 95% CI [2.4, 4.9]. Note, though, that significant heterogeneity of effect size was observed (*Q*(3) = 40.2, *p* < 0.01) due to the unusually small effect observed in Experiment 2 (difficult golf task). Notably, it was this experiment that had the *largest* effect we observed on performance. This figure was generated with the Meta-Analysis module in the Exploratory Software for Confidence Intervals package for Microsoft Excel (Cumming, 2012).

The literature using rated power as a manipulation check for the autobiographical memory task is fairly vast, so we did not attempt a comprehensive meta-analysis. Instead, we used ESCI to integrate results across our studies (Fig 6). We obtained a strong overall effect size (d = 3.7, 95% CI [2.4, 4.9]), though there was significant heterogeneity in effect size due to the unusually weak effect in Experiment 2 (*d* = 2.0). In general, though, the effect sizes we obtained seem well within the range of previous reports. For example, Anderson & Galinsky found an effect size of 2.9 (Study 2, [30]) using a similar procedure with an in-person study and Kifer et al. (Study 2a, [31]) found an effect size of only 1.0 with an large online sample.

## Discussion

We conducted two direct replications and three conceptual replications of Burgmer and Englich’s [2] finding that power increases motor skill. In all five cases, we failed to find a strong effect of power on motor performance. An overall meta-analysis does leave open the possibility that power can have a positive effect on performance. This meta-analysis must be interpreted with caution, however, as it still provides a fairly broad range of estimates, with plausible effect sizes ranging from moderate down to vanishingly small.

### The Perceived-Power Manipulation Check: A Red Flag?

When observed effects are smaller than expected, a likely culprit is insufficient impact from the experimental manipulation. This seems unlikely, though, when considering the rated-power manipulation check: we consistently observed large differences in themes of power in recalled memories, and the effect sizes we observed are generally consistent with previous reports in the literature [3]. Consideration of the perceived-power manipulation check, however, does raise some concerns: this measure revealed more modest and less consistent impact, with both Experiment 1 and 5 failing to produce statistically significant differences on this manipulation check. Moreover, the perceived and rated power measures were only weakly correlated. The perceived-power data, then, could be interpreted as a “red flag” indicative of substantive internal validity issues.

While worthy of consideration, this interpretation is not consistent with the balance of evidence. First, weak manipulation cannot account for our failure to observe strong effects of power on performance. In both Experiments 1 and 5, the sample sizes collected enabled us to conduct reasonably-powered secondary analyses using only participants who had group-appropriate ratings of perceived power. Even though this selection yielded enormous disparities in perceived power, it did not reveal substantive performance differences. Moreover, Experiments 3 and 4 produced large differences in perceived power, but yielded the smallest differences in performance.

There are two factors which may have caused the perceived-power measure to underestimate the true impact of the manipulation. First, we inadvertently adopted a restricted scale (1–5) relative to the original study (0–8) for the measure of perceived power. This may have compressed group differences. Second, and probably more important, perceived power was always measured *after* performance. This sequence was adopted from Burgmer & Englich [2], but it is in contrast to other studies which have used this manipulation check immediately after the memory recall task [23,24,27–29]. Measuring perceived power after performance could have attenuated group differences in two ways: 1) by allowing the passage of time between the memory recall and the manipulation check to decrease the salience of the recalled memory, and 2) by enabling the intervening performance measure to have carryover effects. Consistent with the idea of attenuation over time, the effect size observed by Burgmer & Englich [2] using this sequence was substantially lower than any previous report we could find using the same manipulation and manipulation check (Fig 5). Consistent with the possibility of carryover, we observed a weak correlation between performance and perceived power. Note that in our case, where we did not observe a strong effect of power on performance, carryover from the performance measure would have served to reduce group differences in perceived power. With these factors in mind, the perceived-power data that we obtained does not seem to indicate substantive internal validity problems.

### Why Such Different Effect Sizes?

If our manipulation of power was adequate, why did our experiments yield such meager effects of power on performance compared to Burgmer & Englich [2]? One possible factor could be culture: our participants were U.S. college students whereas Burgmer and Englich [2] studied German college students. Perhaps cultural differences more closely connect power to performance in Europe. Indeed, culture can play a surprisingly large role in even basic psychological phenomenon [32]. One difficulty with this explanation, however, is that the factors proposed by Burgmer and Englich [2] to link power with performance (e.g. greater task striving) do not seem to have any obvious cultural variability between these populations. Indeed, the underpinning research was conducted with British university students [24], American university students (Study 3, [33]), Portuguese university students (Study 1, [33]), and even business people at an international airport [29]. Still, the possibility of a cultural moderator cannot be ruled out. We are currently working on creating a German translation of our online materials to explore if the effect of power on performance can be replicated more robustly in the original cultural setting.

While it is possible to look to experimental impact or moderators to explain why our effect sizes were so small, another perspective is to consider if the effect sizes reported by Burgmer and Englich [2] are unusually large. Theoretically, a large effect of power on performance is puzzling: the results could only have occurred if control participants were performing substantially below their capacity. Burgmer & Englich did not provide any explanation for this large resevoir of latent motor skill. Statistically, it is also important to consider that the precision of an effect size is related to sample size. It is possible, then, that the true effect of the experimental manipulation modest to nil, and that Burgmer and Englich’s [2] results simply reflect the large sampling error associated with small, under-powered experiments (see the confidence intervals for these experiments in Fig 1). Under this interpretation, the true effect size is probably more accurately represented in our results. This would fit what has been called the “winner’s curse” for empirical research [34]: the tendency for extreme estimates of effect size to be prominently published, only to be undermined by subsequent research that uses larger sample sizes to more accurately estimate the true effect size.

### Is it really down to power?

Given the idea of little to no effect on performance, an important consideration is whether or not such results are attributable to the construct of power. The pattern of results in our experiments suggests otherwise. Specifically, group effects on performance did not relate to those observed for perceived power. Large effects on perceived power were sometimes accompanied by minimal differences in performance (Experiments 3 and 4) and one of our largest performance differences occurred with an infinitesimal difference in group power (Experiment 5). The same was true for rated power: the largest performance difference occurred in Experiment 2, which stood out for having an unusually small effect on rated power. This pattern of results suggests that the modest benefits of the experimental manipulation may be unrelated to both perceived and rated power. For example, participants who wrote about a time of power may have simply found the experimental protocol slightly more engaging than those who wrote about a neutral memory, and this could have produced slightly stronger motivation and performance on the motor skills task. Burgmer & Englich [2] examined this possibility in their second experiment (n = 23/group), finding no non-specific effects of manipulating power on mood, arousal, dominance, and self-reported motivation after the word-search priming task (Experiment 2). The sample size used, however, is only adequate to rule out extremely large changes in these confounding variables. If power has a much smaller effect on performance than initially suspected, concerns over subtle, non-specific effects of the manipulation become much more salient. We are currently exploring how manipulating power with the autobiographical recall task influences mood and motivation. Our preliminary results suggest that this manipulation may produce small group differences in excitement that are orthogonal to perceived power. Overall, it is unclear if the non-significant group differences we observed are due to power at all rather than to non-specific effects of the manipulation.

### Replication success?

A secondary goal in conducting this research was to contribute to the empirical record on the replicability of empirical psychology research. Our results, in this respect, are equivocal. Our overall meta-analysis does indicate the possibility that power could have a non-zero effect of performance. In this sense, then, the original finding is vindicated. A more stringent notion of replication, however, may require not only statistical consistency but also a similar level of support for the research conclusions drawn in the original study. In this more expansive sense, our results represent a failed replication: the best current effect size estimate is considerably smaller than envisioned in the original study, and what effect there may be is small enough that it could be accounted for by non-specific effects rather than power.
